# Arm Contouring and Beautification Without Incision: Application of Arm Net Suture

**DOI:** 10.1093/asjof/ojae065

**Published:** 2024-08-23

**Authors:** Sara Ghorbani

## Abstract

**Background:**

Arm contouring has been a challenging issue for many years. Patients request contouring of their arms without incisions and with a shorter recovery time. Making an incision on the arm does not always lead to a nice scar, and in case of complications, it may be catastrophic. The use of “arm net suture” can replace the incision during the arm contour surgery.

**Objectives:**

The author has developed a simple technique of arm contouring with arm net suture that leads to eliminating the need for making an incision, preventing of sagging in the proximal part of the arm, and a shorter recovery time.

**Methods:**

A retrospective study was conducted on 157 patients who underwent arm contouring surgery. Through a small 5 mm incision in the medial elbow, normal saline solution containing Xylocaine (Fresenius Kabi, Bad Homburg, Germany) and adrenaline was infiltrated. After 20 min, liposuction was started from the most superficial layer with a 3 mm cannula, and then, deeper layers superficial to the fascia were suctioned with a 4 mm cannula. The author usually utilizes traditional suction-assisted lipectomy or power-assisted lipectomy. After completing liposuction, the laxity of the skin along the arm was checked. Most of the laxity of the skin occurred in the proximal third of the arm; while the assistant pulled the skin of the proximal part lateral and downward, the net sutures were inserted. On the third or fourth day (based on the severity of laxity of the skin), the sutures start to be removed from the distal rows, with all sutures removed by the fifth to sixth day. The follow-up times were between 3 and 12 months.

**Results:**

All patients were female, and their age range was from 19 to 62 years (mean, 40.45 years). The volume of liposuction was from a minimum of 1200 cc to a maximum of 2500 cc (mean, 1645 cc), and the reduction of the arm diameter was occurred in the range of 6 to 14 cm. No immediate postoperative problems were observed in the patients. Revision was not needed for any patients.

**Conclusions:**

Arm net suture is a simple and safe method to replace the incision during arm contour surgery.

**Level of Evidence: 4:**

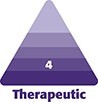

Arm contouring has been a challenging issue for many years. Various classifications have been provided by skilled and experienced plastic surgeons to achieve better results with less complications, by various authors, including Teimourian and Malekzadeh,^1^ Rohrich (in Appelt et al),^2^ and El Khatib.^3^ Various techniques have been developed over the decades to improve contouring, most of which include adding an incision, or changing the location, length, or number of incisions, or introducing different liposuction devices.^4-8^ Patients with massive weight loss (MWL) have attracted considerable attention.^9^ In 2018, Miotto and Ortiz-Pomales^10^ published a complete review article about the evolution of arm contouring. In 2022, Ibrahiem^11^ proposed the use of energy-generating devices in addition to vibration amplification of sound energy at resonance liposuction to remove a scar from the arm.

The author would like to explain the technique that she has utilized for arm contouring surgery and the path that she took to develop a new technique. Since 2010, the author has changed the strategy to create the maximum result with the minimum scar. During the examination, the presence of a step or a lazy horizontal “S” (Sara sign), which is caused by the difference in fat distribution along the length of the arm, was the main factor that determined the surgical method. Therefore, if the step is between one-third proximal and two-thirds distal (Figure 1A), liposuction and the removal of a part of the skin were performed with a transverse incision in the area adjacent to the axilla; however, if the step was in the middle or more distal (Figure 1B), only liposuction was performed. For ∼10 years, there has never been a need for an incision along the length of the arm, and no immediate postoperative problems were observed.

**Figure 1.**
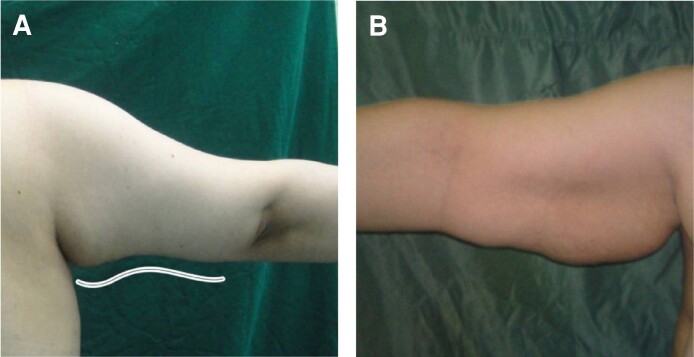
(A) Step sign in the proximal third in a 32-year-old female patient with a BMI 34/m^2^. (B) Step sign in the middle of the arm in a 28-year-old female patient with a BMI 32/m^2^.

In some cases, however, the author encountered mild morbidity that needed time to recover. In the first group, the patients complained of the feeling of skin stretching and limitations in raising their hands. In the second group, ∼8 to 10 cm of the proximal skin would swell and hang because the garment did not fit, thereby creating a constricting band and a pressure effect (Figure 2), and the patient had to utilize a pressure dressing with an elastic band for a long time. Also, in cases without a step along the arm but with a volume of liposuction >900 to 1000 cc, the skin loosening in the proximal half made the need for an elastic band and a garment inevitable for a long time. Another problem, especially in the countries in the author’s region (Middle Eastern Gulf countries), is the lack of acceptance of scars on the arm, especially in young and unmarried girls. Patients often request arm contouring without incisions. Auersvald A and Auersvald LA, 2 Brazilian surgeons, introduced an invention called the “hemostatic net” in 2014, which became the source of inspiration for making changes in arm contouring. The main use of these sutures was in the face and neck to prevent hematoma after facelift surgery.^12^ The purpose of this article is to introduce a technique that eliminates any kind of incision through arm contouring with the aim of creating an arm that has a normal shape and appearance without sagging.

**Figure 2.**
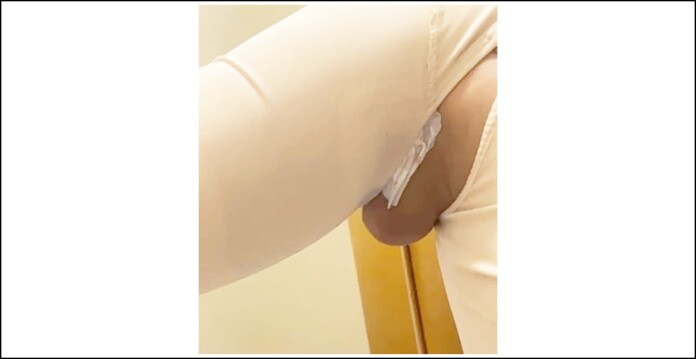
Protrusion and sagging of the skin due to the lack of fitting of the garment and its compressive effect is seen in a 36-year-old female patient with BMI 32/m^2^.

## METHODS

For the previously mentioned reasons, the author has changed her strategy again in 2021. The author planned the redistribution of loose skin after arm liposuction and its stabilization. For this purpose, she utilized sutures to help keep the skin in optimal condition, which she called “arm net suture.” She chose this name because these sutures were neither for hemostasis nor to prevent hematoma, but rather with the support of redistribution of the skin, arm net suture leads to eliminating the need for making an incision to prevent sagging in the proximal part of the arm. The author complied with the principles set forth in the Declaration of Helsinki.

### Patient Selection

Patients with MWL whose sagging part of the arm includes loose skin of poor quality and a small amount of fat deposit are not suitable for this approach. Patients with a BMI >40 are also not suitable candidates because of the surgical risks. For these patients, the author thinks it is better to start contouring the arm after reducing the BMI <40 rather than waiting for the patient to lose a lot of weight or to reach a stable weight. As a result, there is no need for brachioplasty with long incision in many cases.

Except 2 groups of patients with MWL and patients with BMI >40, every patient who is referred due to arm obesity and sagging is selected for surgery. Certainly, underlying diseases and smoking should be considered, but because an incision is not utilized, the restrictions in the presence of these diseases are less. The patient stands or sits on the bed with the arm placed at a 90° angle of abduction. The quality of the skin and the amount of fat are also evaluated.

### Markings

**Figure 3.**
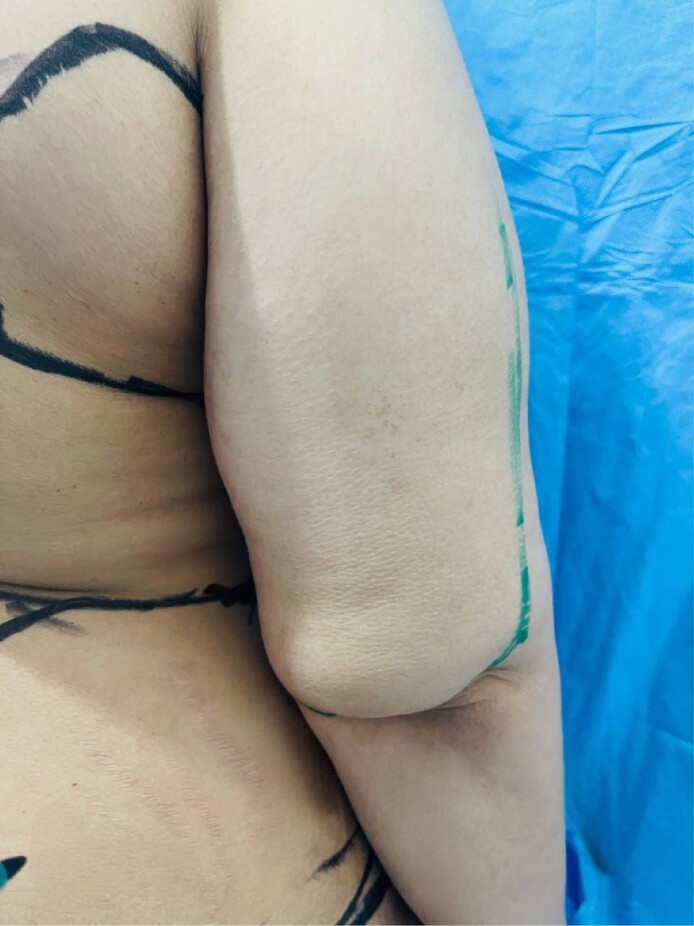
The fat pad above the elbow in a 46-year-old female patient with BMI 36/m^2^ is seen when the arm is in the rest position.

Before starting the marking, the arm circumference is measured in the area that seems to have the largest diameter, which is often in the proximal third. Arm size is not an effective criterion by itself in determining the surgical plan, but comparing the size before and after surgery and knowing the amount of diameter reduction should satisfy both the patient and the surgeon. The most amount of fat deposition occurs in the posterior and somewhat medial parts. The patient stands facing the wall, the surgeon is behind the patient, and the patient places the arm by the side of the body in the resting position. Usually, there is a fat pad just above the elbow, which is only visible when the arm is in this position and disappears when the arm is in the abduction position (Figure 3). Marking starts around this fat pad and extends proximally. Then, the arm is placed in the abducted and horizontal position, first at the back, then with the rotation of the patient, the marked boundaries are checked and completed so that they include all of the hanging parts, or vice versa. The marking can be started in a horizontal position, then placed by side, and then the fat pad above the elbow is identified and included. Also, if there is a fat pad in the superolateral part of the arm, it is marked (Video 1).

### Procedure

If the contour of the arm will be combined with other surgeries in the posterior areas of the body, the patient is placed in the prone position; if arm contouring will be done by itself, the patient is placed in the supine position. The preferred method for anesthesia is general anesthesia, but in some cases, if the patient cooperates, the procedure can be performed under local anesthesia and IV sedation. After the induction of anesthesia, the arms are prepared and the hands and forearms are covered up to the elbows. The author infiltrates solution, which is normal saline containing adrenaline and Xylocaine (lidocaine HCl) (Fresenius Kabi, Bad Homburg, Germany), through a small 5 mm incision on the medial side above the elbow. One or 2 more holes may be required to access all parts. The author usually infiltrates until the tissues are tense, with mean infiltration to the extent of swelling and tensing of the skin. After 20 min, liposuction will be started from the most superficial layer adjacent to the skin with a 3 mm cannula and then proceeded to the deeper layers superficial to the fascia with a 4 mm cannula. All of the thickness of the fat is typically superficial to the muscle and its fascia. Neurovascular vessels are not in this area and are protected by muscles. The sagging part usually contains the fat deposit.

The author utilizes traditional suction-assisted lipectomy or power-assisted lipectomy (PAL) (Video 2). For someone who has less experience in superficial liposuction, PAL is better because it reduces the risk of creating irregularities. However, the type of liposuction device is not the criterion for obtaining better results, thus plastic surgeons can utilize any device that is more comfortable and provides them with better results. Liposuction is completely performed in the desired areas. If the fat deposition was in the superolateral part, which led to the step, it was also suctioned. After completing liposuction, the laxity of the skin along the arm was checked. There was usually a clear difference in the amount of laxity of the skin along the length of the arm. The lowest level of laxity occurred in the distal half to distal third. It is better to apply sutures along the entire length of the area with maximum laxity.

**Figure 4.**
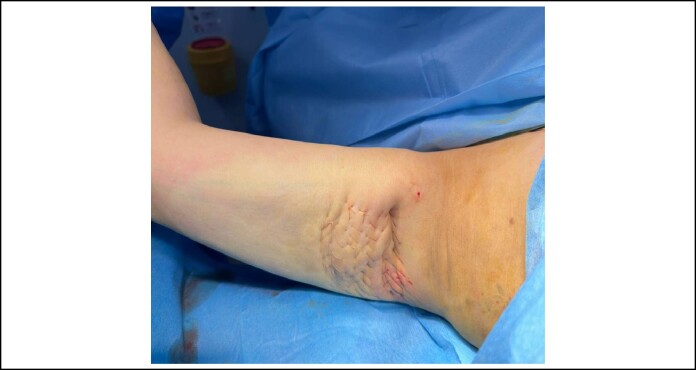
Arm net sutures in area about 8 cm with skin laxity after liposuction in a 36-year-old female patient with BMI 30/m^2^.

In her experience, the biggest problem was in the proximal 8 to 10 cm. While the assistant pulled the skin of the proximal part lateral and downward, the net sutures were inserted (Video 3). Usually, all types of threads, either 3 zero or 4 zero, with a needle 30 mm or larger, are utilized. The skin of the arm is thick, so the needle size is important because it must cover the full thickness of the skin as well as the underlying fascia. It is not necessary to include the fascia in all sutures; utilizing 2 or 3 points of suture fixation to the fascia in each row is sufficient. It is better that the sutures are not completely continuous and are done in separate double rows (Figure 4). After finishing the sutures, the arms should be cleaned, and antibiotic ointment should be applied to the area of the sutures. Then, the arm garment, which is not very tight, is worn.

### Postoperative Care

On the third or fourth postoperative day (based on the severity of the laxity of the skin), removal of sutures was begun from the distal rows. By the fifth to sixth postoperative day, all sutures are removed, even though the garment continues to be utilized. After 3 weeks, when the swelling and pain subside, it is better to use a smaller and tighter size. The author recommends using the garment during 24 h/day for 4 to 6 weeks. During the day for the next 4 to 6 weeks, wearing a garment is important from a psychological aspect, and in patients who have a high volume of liposuction, it is also useful for reducing edema and skin shrinkage. Increasing or decreasing the period of using the garment depends on the volume of liposuction and the degree of skin laxity, thus it is optional. If the suture marks are still seen after their removal, a brightening cream containing hydroquinone or retinol would be prescribed. After 2 to 3 weeks, the suture marks will disappear.

## RESULTS

**Figure 5.**
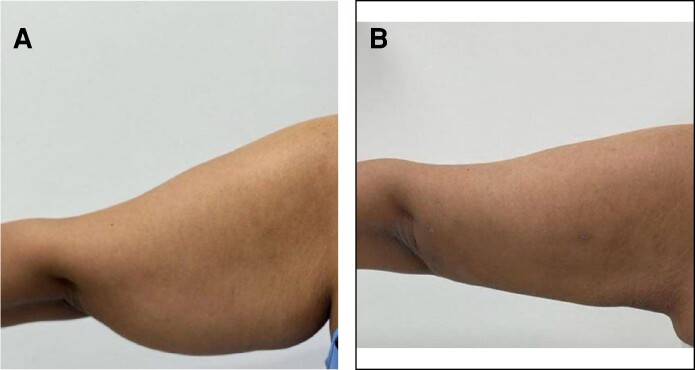
A 43-year-old female patient with BMI 36/m^2^. (A) Preoperative and (B) 6 months postoperative photographs after liposuction and net sutures. The diameter reduction is about 14 cm and the amount of suction volume for each arm was about 1100 cc.

**Figure 6.**
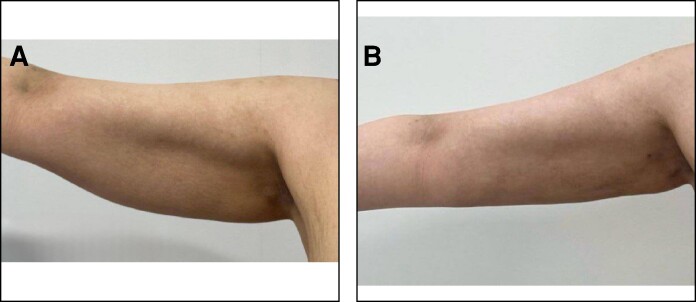
A 35-year-old female patient with BMI 30/m^2^. (A) Preoperative and (B) 11 months postoperative photographs after liposuction and net sutures. The diameter reduction is about 7 cm and the amount of suction volume for each arm was about 600 cc.

**Figure 7.**
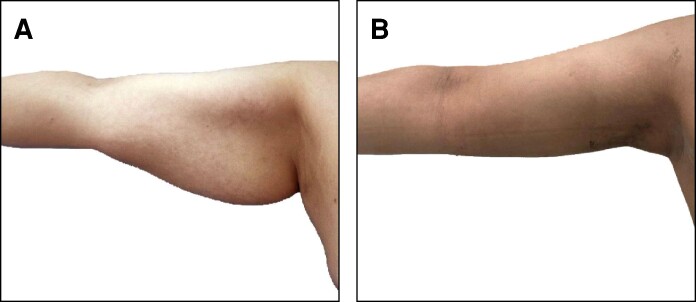
A 38-year-old female patient with BMI 35/m^2^. (A) Preoperative and (B) 9 months postoperative photographs after liposuction and net sutures. The diameter reduction is about 9 cm and the amount of suction volume for each arm was about 800 cc.

The author performed the arm net suture technique without incision on 157 patients. The follow-up times were from 3 to 12 months. All patients were female, and their age range was from 19 to 62 years (avg., 40.45 years). The volume of liposuction is from a minimum of 1200 cc to a maximum of 2500 cc (avg., 1645 cc), and the reduction of the arm diameter has occurred in the range of 6 to 14 cm. No immediate postoperative problems were observed in the patients. Revision was not needed in any case (Figures 5-7).

## DISCUSSION

There are no defined criteria for a beautiful contour of the arms. In fact, there is no accurate information about which arm shape is called beautiful. Athletes’ arms with hypertrophied muscles that show the limits of anatomy may be considered beautiful, but this model is not ideal for many people, particularly females. People who come for arm contour often request that the sagging and looseness of the skin be reduced in size. Usually, the person says that the size of the arm is not proportional to the rest of the body, so it is difficult to choose clothes. Based on the patient's complaint, the plastic surgeon decides to perform surgery with the aim of improving the contour of the arms and reducing their size. Various classifications have been provided by skilled and experienced plastic surgeons to achieve better results with less complications. Usually, MWL patients attract attention. Therefore, the focus is on creating an incision and performing an open surgery.

In many patients with MWL, there may not be a reduction in the size of the arm proportional to the rest of the body, but the obesity remains and the sagging increases. On the other hand, arm obesity and sagging may exist in isolation, which causes disproportion compared with the size for the rest of the body and causes associated difficulties, especially when buying clothes. Usually, in generalized obesity, the size of the arm is at the center of the patient's attention. Brachioplasty is the chosen plan in most cases, unless the skin quality is very good, and when a size reduction of 3 to 4 cm is considered, liposuction can be performed without incision. The development of technology and the use of radiofrequency have led to a more courageous treatment for the contour of the arm; however, first, these technologies are not available to everyone, and second, they do not eliminate the need to create an incision in all sagging cases. Nowadays, due to the wide range of advertisements in social media and some strange results after surgery, which are difficult for us as plastic surgeons to believe, we are forced to improve the quality of surgical procedures based on the expectations of patients and society.

Patients who come to beautify the arms do not want to accept another problem: scar formation. Beauty is not only reserved for the face but also applies to the arms. In the case of the face, the surgical incision can be hidden, but in the case of the arms, any type of incision is completely visible, and in cases which the incision becomes complicated, it may lead to mediocre results. What is shown from the brachioplasty scar view in the social media, books, and articles is a very narrow and hard-to-see line, while the terrible scars and complications are often hidden and not shown. Arm contouring is one of the most challenging aesthetic surgeries; hence, plastic surgeons may be reluctant to accept these patients. Performing abdominoplasty or mammaplasty is more favorable for a plastic surgeon, and the related scars are easily accepted by the patient. For many years, the author has tried to improve the results of arm contouring and reduce complications and morbidity.

During liposuction in any part of the body, at the same time that the fat deposit is removed, the skin is given a new nature and the distribution of the skin in the area is redefined. It is the same with the arm after liposuction, when there is skin with a new nature and quality that needs to be redistributed. The net sutures should be utilized to help keep the redrape skin in optimal condition. The main cornerstone of arm contouring is liposuction, and in order to maintain the redistribution of the skin with a new nature, the use of arm net suture replaces the longitudinal incision along the length of the arm or the transverse incision in the axilla region. The use of net suture in the length of the arm is versatile, and its extent depends on the extent of laxity of the skin. According to the author’s experience, the most prone part to laxity is the proximal third of the arm, especially its initial 8 to 10 cm, and usually the use of net sutures in this area is sufficient.

In addition to the use of net sutures, it should also be explained to the patient before the operation that it takes time for the skin to shrink and tighten and to adapt to the new foundation. This period requires patience from both the surgeon and the patient. During the study, fortunately, the patients easily accepted the use of net suture.

During this work, an important limitation was the unavailability of a suitable needle and thread. For this procedure, a fine thread is required to be attached to a large size and, of course, a fine needle so that the entrance points become invisible.

## CONCLUSIONS

With the correct technique of liposuction and by fixing the redistributed skin in proper condition with the use of the arm net suture, it is possible to beautify and contour the arms without incision, which can achieve satisfactory results without complications or the need for revisions.

## References

[1] Teimourian B, Malekzadeh S. Rejuvenation of the upper arm. Plast Reconstr Surg. 1998;102(2):545–551; discussion 552. doi: 10.1097/00006534-199808000-00041 9703097

[2] Appelt EA, Janis JE, Rohrich RJ. An algorithmic approach to upper arm contouring. Plast Reconstr Surg. 2006;118(1):237–246. doi: 10.1097/01.prs.0000231933.05534.95 16816702

[3] El Khatib HA. Classification of brachial ptosis: strategy for treatment. Plast Reconstr Surg. 2007;119(4):1337–1342. doi: 10.1097/01.prs.0000254796.40226.92 17496609

[4] El-Fahar MH, El-Gharbawi AH. Ultrasound-assisted liposuction (UAL) arm contouring in non-post-bariatric patients: no rush for brachioplasty. Aesthet Plast Surg. 2023;47(1):260–268. doi: 10.1007/s00266-022-03070-8 PMC9944711 36042028

[5] Rohrich RJ, Mohan R, Durand PD. Brachioplasty refinements. Plast Reconstr Surg. 2020;145(4):754e–756e. doi: 10.1097/PRS.0000000000006688 32221211

[6] Sergesketter AR, Erdmann D. A personal approach to brachioplasty. Ann Plast Surg. 2022;88(5 Suppl 5):S433–S438. doi: 10.1097/SAP.0000000000003119 35226447

[7] Leclère FM, Alcolea JM, Vogt PM, et al Laser-assisted lipolysis for arm contouring in Teimourian grades III and IV: a prospective study involving 22 patients. Plast Surg (Oakv). 2016;24(1):35–40. doi: 10.1177/229255031602400105 27054137 PMC4806755

[8] Nguyen AT, Rohrich RJ. Liposuction-assisted posterior brachioplasty: technical refinements in upper arm contouring. Plast Reconstr Surg. 2010;126(4):1365–1369. doi: 10.1097/PRS.0b013e3181ebe23c 20885260

[9] Myers PL, Bossert RP. Arm contouring in the massive-weight-loss patient. Clin Plast Surg. 2019;46(1):85–90. doi: 10.1016/j.cps.2018.08.011 30447832

[10] Miotto G, Ortiz-Pomales Y. Arm contouring: review and current concepts. Aesthet Surg J. 2018;38(8):850–860. doi: 10.1093/asj/sjx218 29546270

[11] Ibrahiem SMS. Aesthetic nonexcisional arm contouring. Aesthet Surg J. 2022;42(7):NP463–NP473. doi: 10.1093/asj/sjac031 35170726

[12] Auersvald A, Auersvald LA. Hemostatic net in rhytidoplasty: an efficient and safe method for preventing hematoma in 405 consecutive patients. Aesthet Plast Surg. 2014;38(1):1–9. doi: 10.1007/s00266-013-0202-5 23949130

